# Bed rest and endurance retraining alter persistent inward current estimates in young and older males

**DOI:** 10.3389/fphys.2026.1905824

**Published:** 2026-09-22

**Authors:** Giovanni Martino, Miloš Kalc, Maíra Camargo Scarpelli, Fabio Sarto, Mohammadhossein Ghasemi, Riccardo Magris, Rado Pišot, Boštjan Šimunič, Nina Murks, Ales Holobar, Simone Porcelli, Martino V. Franchi, Marco V. Narici, Giuseppe De Vito

**Affiliations:** 1 Department of Biomedical Sciences, University of Padova, Padova, Italy; 2 Institute for Kinesiology Research, Science and Research Centre Koper, Koper, Slovenia; 3 Faculty of Electrical Engineering and Computer Science, University of Maribor, Maribor, Slovenia; 4 Department of Molecular Medicine, Institute of Physiology, University of Pavia, Pavia, Italy; 5 CIR-MYO Myology Center, University of Padova, Padova, Italy

**Keywords:** aging, endurance training, high-density electromyography, motor unit, muscle disuse

## Abstract

**Introduction:**

Prolonged physical inactivity induces rapid neuromuscular deconditioning, with aging influencing both the rate, recovery and nature of these changes. However, its effects on the modulation of motoneuron excitability across age groups remain unclear. This study examined how bed rest and subsequent endurance retraining influence persistent inward current (PIC)-related estimates, motor-unit (MU) discharge behavior, and circulating tryptophan (TRP) and kynurenine (KYN) biomarkers in healthy young and older males.

**Methods:**

Nine young (22 ± 4 yr) and ten older (69 ± 3 yr) males completed 21 and 10 days of horizontal bed rest, respectively, followed by 21-day endurance retraining. High-density surface EMG decomposition of vastus lateralis was used to estimate PIC-related indices derived from paired MU analysis (ΔF) and geometric discharge metrics, namely brace height (BH), acceleration (ACC), and attenuation (ATT), as well as MU discharge properties, including peak discharge rate and discharge-rate ranges. Plasma TRP, KYN, and their concentration ratio were also quantified.

**Results:**

Bed rest reduced ΔF in YA after 21 days (-26.1%, p<0.001) and in OA after 10 days (-15.3%, p=0.029). After retraining, ΔF was no longer different from baseline in YA, whereas in OA values at RT did not differ from either baseline or bed rest. In YA, ACC (-25.1%, p=0.002) and ATT (-27.7%, p=0.023), but not BH, decreased after bed rest and were no longer different from baseline after retraining; in OA, BH, ACC, and ATT were unchanged. Peak discharge rate and discharge-rate range decreased after bed rest in both groups and showed partial recovery after retraining. Plasma tryptophan, kynurenine, and their ratio did not change.

**Discussion:**

These findings indicate that motoneuron-related indices are sensitive to prolonged physical inactivity and remain modifiable during post-disuse retraining. Although older adults were exposed to a shorter bed rest duration, ΔF adaptations to disuse and retraining were also present in an older population. The non-uniform behavior of ΔF and geometric discharge metrics suggests that different aspects of motoneuron output may adapt through partly distinct mechanisms, potentially with age-dependent features.

## 1 Introduction

Bed rest is a well-established model of prolonged physical inactivity (Pavy-Le Traon et al., 2007), replicating conditions observed during hospitalization, injury recovery, or spaceflight (Narici and de Boer, 2011). It induces rapid declines in muscle mass, strength, and functional capacity (Kortebein et al., 2008; Marusic et al., 2021), and provides a controlled framework to investigate the mechanisms underlying deconditioning in both young and older adults (Murgia et al., 2022). At the peripheral level, disuse leads to muscle fiber atrophy (Trappe et al., 2004), impaired excitation-contraction coupling and neuromuscular transmission efficiency (Duchateau, 1995; Monti et al., 2021; Sarto et al., 2025), while motor unit (MU) discharge exhibits greater variability and reduced rate (Duchateau and Hainaut, 1990; Inns et al., 2022; Sarto et al., 2022). Although these adaptations diminish the capacity for rapid and sustained force production, they are largely reversible through exercise (Suetta et al., 2009; Valli et al., 2024; Martino et al., 2024; Franchi et al., 2025). In contrast, less is known about how disuse and subsequent recovery influence the central neural mechanisms governing motoneuron excitability.

Persistent inward currents (PICs) are voltage-dependent depolarizing currents generated in motoneuron dendrites and facilitated by monoaminergic neuromodulatory input. PICs are key determinants of motoneuron excitability shaping motor unit discharge in three characteristic ways: amplifying excitatory synaptic input producing an initial acceleration of discharge rate; saturating thereafter, attenuating the subsequent rate rise; and prolonging firing below the excitatory drive required for recruitment, generating onset–offset hysteresis (Heckman et al., 2008; Beauchamp et al., 2023). Because discharge rate reflects the combined action of excitatory, inhibitory and neuromodulatory inputs, which jointly determine the shape of the motoneuron input–output function (Powers and Heckman, 2017), these contributions cannot be disentangled from motor unit output alone, and several complementary estimation techniques have been developed. The most widely used is the paired motor unit analysis (Gorassini et al., 2002), in which the difference in discharge rate of a lower-threshold reporter unit between recruitment and derecruitment of a higher-threshold test unit (ΔF) indexes the prolongation component of PIC activity, although it is sensitive to the pattern of inhibitory input as well as to neuromodulatory drive (Hyngstrom et al., 2008; Johnson et al., 2012). More recently, a geometric approach quantifying the non-linearities of individual discharge profiles brace height, acceleration slope, attenuation slope and angle has been proposed (Beauchamp et al., 2023): in a simulated motoneuron pool, brace height tracked neuromodulation with comparatively little dependence on inhibitory pattern, whereas attenuation slope was informative about excitation–inhibition coupling. Together, these approaches provide complementary access to the amplification and prolongation components of PIC activity.

We recently showed that short-term disuse via unilateral lower-limb suspension reduces ΔF in healthy young adults (Martino et al., 2024), and that active recovery can restore these PIC-related estimates (Martino et al., 2024). It is unknown whether prolonged whole-body bed rest produces similar changes in young and older adults. Previous work has suggested that aging may be associated with lower PIC estimates and reduced motoneuron excitability (Orssatto et al., 2023), which could increase susceptibility to neuromuscular deconditioning during periods of inactivity. However, the effects of bed rest on PIC-related estimates in young and older adults, and their response to retraining, have not been investigated.

In addition to electrophysiological measures, circulating biomarkers may provide complementary systemic context for the physiological response to disuse and retraining. Because serotonergic input is known to facilitate PIC activation in motoneurons (Perrier et al., 2013), indices related to serotonin synthesis may be of exploratory interest. Accordingly, circulating tryptophan (TRP), kynurenine (KYN), and their ratio may provide indirect insight into systemic serotonergic availability (Hinkley et al., 2023) and its potential influence on MU behavior.

Therefore, we investigated the effects of prolonged bed rest and subsequent endurance retraining on PIC-related estimates in young and older males. We hypothesized that bed rest would reduce PIC-related measures and that retraining would promote recovery. We explored whether motor-unit discharge behavior changed alongside PIC-related indices and whether these changes were accompanied by alterations in circulating TRP, KYN, and their ratio.

## 2 Materials and methods

### 2.1 Participants

We recruited nine young healthy adults (YA; mean ± SD: age 22.8 ± 4.4 years; height 1.83 ± 0.06 m; body mass 79.6 ± 6.8 kg) and ten older healthy adults (OA; age 68.5 ± 2.6 years; height 1.73 ± 0.07 m; body mass 86.8 ± 12.3 kg). Bed rest studies are constrained by requirements for medical supervision, cost, and logistics, which limit achievable sample sizes. Nonetheless, an *a priori* power analysis was performed using expected effect sizes derived from previous bed rest studies reporting losses in muscle mass and force in young (Suzuki et al., 1994; Kawakami et al., 2001) and older participants (Pišot et al., 2016). With α = 0.05 and a target power of 0.70, accepted *a priori* in view of the constraints described above, 10 participants were required.

To our knowledge, no previous bed rest study has quantified PIC-related estimates in humans; therefore no prior effect size was available for a formal sample size estimation for these outcomes. However, in our previous study employing an unloading model of a similar duration, 10 days of unilateral lower limb suspension were sufficient to produce a significant reduction in the ΔF of vastus lateralis motor units in 12 young adults (Martino et al., 2024).

Only male participants were included in the present study, as participation of females was not permitted under the ethics committee approval for this bed-rest protocol; therefore, the findings cannot be generalized to females. Participants underwent a comprehensive medical screening prior to inclusion. Exclusion criteria encompassed cardiovascular, musculoskeletal, neuromuscular, metabolic and psychiatric conditions, alongside smoking, drug use, professional sports involvement and any contraindications identified during the medical screening. The study adhered to the Declaration of Helsinki and was approved by the National Ethical Committee of the Slovenian Ministry of Health (0120-123/2023/9). Written informed consent was obtained from all participants, who were informed of their right to withdraw at any time.

### 2.2 Experimental design

Baseline data collection (BDC) was performed 2 days before bed rest, and assessments were repeated on the first day of recovery (BR21 for YA, BR10 for OA) and after 21 days of active retraining (RT) (**Figure 1**).

**Figure 1:**
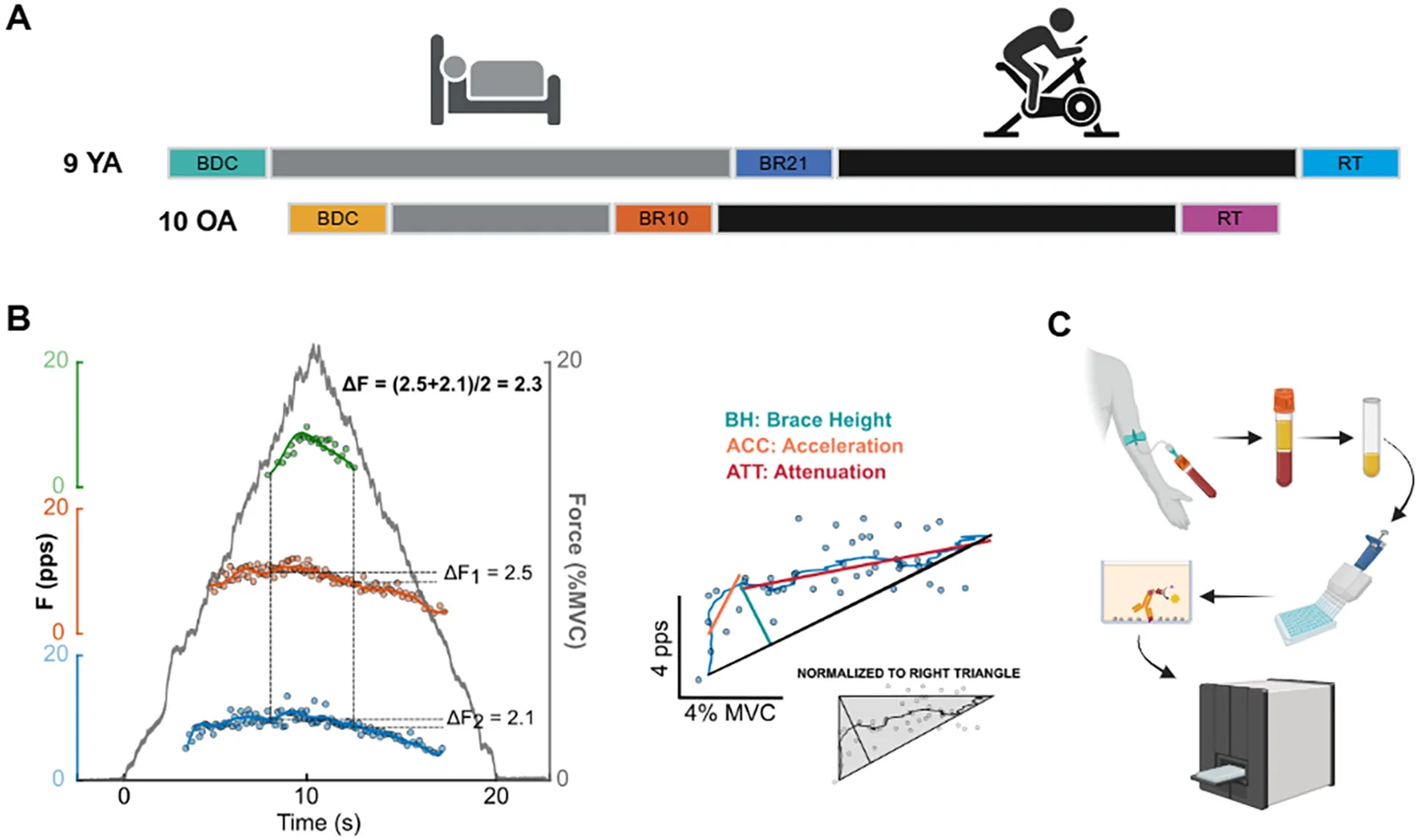
Study design **(A)** Nine healthy young adults (YA) and 10 healthy older adults (OA) participated in this study. Experimental measurements were collected at baseline (day 0 prior bed rest, BDC), after 21 days of bed rest (BR21) for YA and after 10 days of bed rest (BR10) for OA and following 21 days of retraining (RT). **(B)** High density surface electromyography (HDsEMG) from vastus lateralis muscle and force profiles were collected during isometric triangular contractions at 20% of maximal voluntary contraction. From decomposed motor units, estimates of persistent inward currents (ΔF) were computed using the paired motor unit technique (left). Additionally, a geometric approach (right) was applied to the discharge rate-force relationship of individual motor units to extract 3 metrics: brace height, acceleration and attenuation slope. **(C)** At each data collection point blood samples were collected from medial cubital vein. Samples were then centrifuged to separate blood components. Following, plasma was prepared into 100 µl aliquots and stored at -80 °C until processing. Tryptophan and Kynurenine plasma concentrations were then obtained using enzyme-linked immunosorbent assay (ELISA) analysis. Created with BioRender.com.

#### 2.2.1 Bed rest

YA underwent 21 days of horizontal bed rest, whereas OA underwent 10 days. The 21-day duration in YA was predefined as part of the broader study protocol and was selected to provide a robust whole-body disuse stimulus sufficient to induce measurable neuromuscular adaptations under continuous medical supervision (Marusic et al., 2021). In contrast, the OA protocol was limited to 10 days because of ethics committee restrictions related to the increased cardiovascular risk associated with prolonged inactivity in older participants (Mastrandrea et al., 2024). This resulted in unmatched disuse durations between groups, and direct age comparisons should therefore be interpreted cautiously. The study took place in August–September 2023 at the General Hospital of Izola, Slovenia, under 24-hour medical supervision, with video monitoring to ensure compliance. All daily activities were performed in a strictly horizontal position, and no voluntary exercise or systematic movements were permitted. Participants received a eucaloric diet consisting of 60% carbohydrate, 25% fat, and 15% protein, provided 4 times daily, with water and herbal tea available ad libitum.

#### 2.2.2 Retraining

Immediately after bed rest, participants began a 3-week supervised endurance retraining program consisting of high-intensity interval training performed on a cycle ergometer 3 times per week. Sessions were structured at an average intensity of 80% of the post-bed rest gas exchange threshold, determined using the modified V-slope method (Beaver et al., 1986). The total weekly volume provided approximately 150 minutes of moderate-to-vigorous activity, in line with WHO recommendations (Bull et al., 2020). Endurance retraining was selected as a clinically relevant post-disuse intervention (Lee et al., 2010; Vigelsø et al., 2015) to examine whether a structured aerobic exercise stimulus is sufficient to promote recovery of neuromuscular function after inactivity. Endurance-based exercise may be particularly relevant in the early phase of recovery after prolonged bed rest, when high-load resistance exercise may not yet be appropriate or well tolerated.

### 2.3 Experimental protocol

#### 2.3.1 Force and HDsEMG recording

Maximal voluntary contraction (MVC) of the knee extensors of the right leg was measured at each data collection point using an isometric dynamometer (RS 206-0290; Tedea Huntleigh, Selb, Germany) with the knee and hips at 90°. Following a warm-up of ten submaximal contractions (50–90% of perceived maximum), 3 MVC trials were performed with 1-minute rest intervals, with visual feedback and verbal encouragement provided throughout. The highest force value across trials was considered MVC.

Participants then performed 3 triangular isometric contractions at 20% of MVC, each lasting 20 seconds with ramp-up and ramp-down rates of 2% MVC per second. The 20% MVC target was selected to facilitate accurate HDsEMG decomposition during stable low-force contractions, while providing a physiologically relevant intensity for daily functional tasks involving the knee extensors (Tikkanen et al., 2013; Marshall et al., 2020). To account for possible changes in absolute force across time points (Orssatto et al., 2021), 3 additional triangular contractions at 20% of baseline MVC were also collected post-bed rest and post-retraining; analyses based on these contractions are reported in the **Supplementary Materials**. Force signals were synchronized with high density electromyography (HDsEMG) signals sampled at 2048 Hz using a multichannel amplifier (Quattrocento, OT Bioelettronica, Torino, Italy) and low-pass filtered bidirectionally before analysis (20 Hz, 4th order, zero-phase Butterworth filter).

HDsEMG was recorded from the vastus lateralis muscle (VL) using a 64-channel grid (GR08MM1305, OT Bioelettronica) positioned over the distal innervation zone, approximately 30% of femur length. Before electrode placement, the skin over the recording site was shaved and lightly abraded using an abrasive-conductive paste (Spes Medica, Salerno, Italy). The grid was filled with conductive paste (Ten20; Weaver and Company, Aurora, CO, USA) and aligned with fascicle orientation determined by B-mode ultrasonography. The central electrode of the penultimate row was positioned over the innervation zone identified by low-intensity percutaneous stimulation as the location requiring the lowest stimulation current to evoke a visible muscle twitch. Reference and ground electrodes were placed on the patella and lateral malleolus, respectively.

#### 2.3.2 HDsEMG analysis

Monopolar HDsEMG signals were decomposed offline in MATLAB (R2023a, MathWorks) using the Convolution Kernel Compensation (CKC) algorithm (Holobar and Zazula, 2007) implemented in DEMUSE (v6, Maribor, Slovenia). Before decomposition, HDsEMG channels were screened for outliers by calculating the average pairwise correlation coefficient between each channel and its directly neighboring channels. Channels with an average correlation coefficient below Q1-1.5xIQR were removed, with a maximum of five channels excluded per recording. The remaining HDsEMG signals were then band-pass filtered bidirectionally (20–500 Hz, 2nd order, zero-phase Butterworth filter). Signals from the 3 triangular contractions within each assessment were concatenated and decomposed jointly, so that the same decomposition filters were applied across all contractions within a session, facilitating consistent identification of MUs within that session. MUs were not tracked across experimental sessions.

Identified MUs were visually inspected and manually refined to correct missed, duplicated, or artefact-related discharge events when necessary. Pulse to Noise Ratio (PNR) was used as a signal-quality metric, with 30 dB considered the reference threshold for reliable decomposition (Holobar et al., 2014). Units with PNR values below this threshold were inspected carefully, and retained only when visual inspection confirmed clearly separable discharge events from other units, physiologically plausible inter-spike intervals, and stable discharge patterns consistent with the triangular force trajectory. All retained MUs were carefully inspected for irregular inter-spike intervals and implausible discharge patterns. When necessary, missed or artefact-related MU discharge events were manually added or removed by an experienced operator. Discharge rates were computed as the inverse of inter-spike intervals. Recruitment threshold was defined as the force level at the first identified discharge of each MU, and derecruitment threshold as the force level at the last identified discharge. Peak discharge rate was calculated from the discharge profile as the maximal discharge rate reached during the contraction. Discharge rate range was defined as the difference between peak discharge rate and recruitment discharge rate. Ascending and descending discharge rate ranges were defined as the difference between peak discharge rate and recruitment or derecruitment discharge rate, respectively.

To quantify the contribution of PIC-related behavior to motoneuron discharge, we applied paired MU analysis (Gorassini et al., 2002) and a complementary geometric approach (Beauchamp et al., 2023). Before paired MU and geometric analyses, instantaneous discharge rates were smoothed using support vector regression (SVR; fitrsvm function in Matlab), as previously described by Beauchamp et al. (2023), to reduce discharge variability and obtain continuous discharge profiles. Specifically, SVR used a radial basis function kernel (kernel scale = 1.6), with epsilon set to one-eleventh of the discharge-rate interquartile range, and a box constraint of 370. The first and last five discharge events were weighted fivefold relative to the remaining events to improve fitting at recruitment and derecruitment.

Paired MU analysis: PIC-related estimates were derived by quantifying onset-offset hysteresis (ΔF) in the discharge rate of a higher-threshold MU (test unit), expressed relative to the concurrent discharge rate of a lower-threshold unit (control unit). MU pairs were included when the following criteria were met (Martino et al., 2024): (1) test unit recruitment occurred at least 1 s after control unit recruitment; (2) control unit derecruitment occurred at least 1.5 s after test unit derecruitment; (3) control unit discharge-rate modulation, defined as the range of its smoothed discharge rate while the test unit was active, exceeded 0.5 pulses per second; (4) Pearson’s rate-rate correlation coefficient between the smoothed discharge-rate profiles of the test and control units over their period of concurrent discharge was r > 0.7. ΔF was calculated for each valid pair as the difference between control unit discharge rates at test unit recruitment and derecruitment, providing an index of the contribution of PICs to self-sustained firing (Afsharipour et al., 2020). ΔF was calculated independently for each valid test-control MU pair within each triangular contraction. When a test unit was paired with multiple valid control units within each contraction, ΔF values were averaged to obtain one “unit-wise” ΔF value for that test unit.

Geometric analysis: Brace height (BH) was defined as the maximum orthogonal deviation between the smoothed discharge rate and an expected linear increase from recruitment to peak discharge, normalized to the height of a right triangle (%rTri) with a hypotenuse spanning from recruitment to peak discharge (Beauchamp et al., 2023). Additionally, slopes for the acceleration (ACC) and attenuation (ATT) phases of MU discharge were computed to distinguish between secondary and tertiary discharge phases. MUs with a negative ACC, BH exceeding 200%rTri, or peak discharge occurring after peak force were excluded from analysis.

#### 2.3.3 Blood sampling

Fasted blood samples were obtained from the medial cubital vein at the same time of day at each timepoint. Samples were stored at room temperature for 10 min and then centrifuged (CENTRIC 400, Domel, Železniki, Slovenia) at 2500g (3880 rpm) to separate blood components. Subsequently, plasma was prepared into 100 µl aliquots, which were immediately frozen in liquid nitrogen and stored at -80 °C until processing. Enzyme-linked immunosorbent assay (ELISA) analyses were carried out to quantify plasma levels of TRP (cat. no. BTB-E4244hu; BT Lab, Zhejiang, China) and KYN (cat. no. BA E-2200R; Immusmol, Bordeaux, France). A microplate ELISA reader (450nm; Tecan, Infinite M200, Männedorf, Switzerland) was used to read absorbance at 450 nm. The plasma concentrations of each marker were then obtained by interpolation from the respective standard curve and corrected for sample dilution when necessary. All samples were analyzed in duplicate and the average coefficients of variation for the measurements were 4.3% and 3.0%, respectively.

### 2.4 Statistical analysis

All statistical analyses were performed using R Statistical Software (v4.5.3; R Core Team 2026). Differences in MVC and plasma concentrations of TRP and KYN were examined using repeated-measures ANOVA, followed by Bonferroni-corrected paired t-tests for *post hoc* comparisons. PIC-related variables and MU discharge characteristics were analyzed using separate linear mixed models per variable and age group. Time was included as a fixed effect. Subject was included as a random intercept to account for repeated measurements within individuals. A second random intercept for MUs nested within subject and session was included to account for clustering of multiple identified units within each participant at each assessment and for unequal numbers of MUs across participants and sessions. MUs were not tracked across sessions and were therefore treated as independent within each time point. Model assumptions were assessed graphically using Q-Q plots, residual-versus-fitted plots, homoscedasticity checks, and inspection of influential observations. Pairwise comparisons between time points were performed using estimated marginal means with Bonferroni correction. The significance threshold for all analyses was set at α = 0.05.

## 3 Results

### 3.1 Maximal voluntary contraction force

In YA, 21 days of bed rest affected the maximal knee extensor force (**Figure 2A**). MVC decreased at BR21 compared to BDC (23.5%, p<0.001) and remained lower at RT than at BDC (15.4%, p=0.01), although MVC increased from BR21 to RT (11.7%, p=0.010), indicating partial recovery after retraining. In OA, MVC force did not differ across time points (**Figure 2B**).

**Figure 2:**
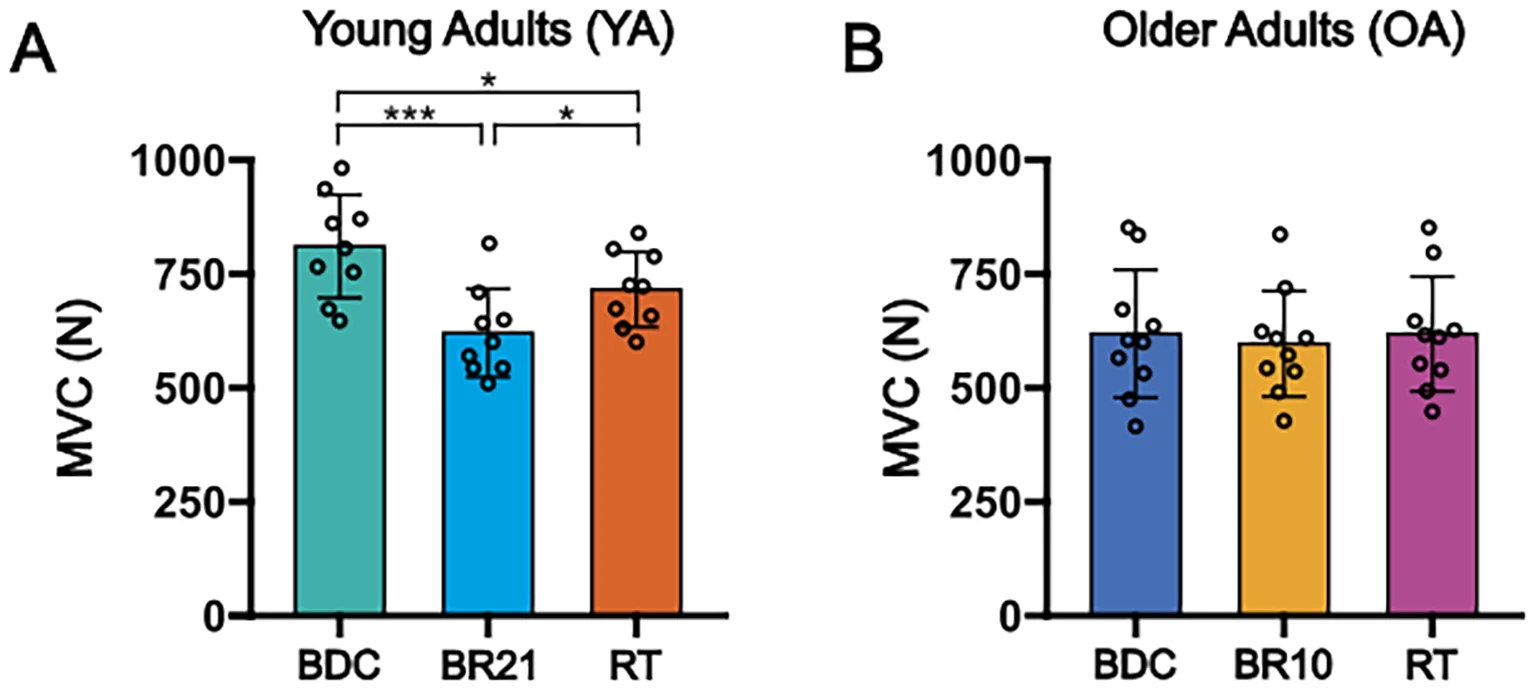
Bar plots show MVC force measured at BDC, BR21 and RT for YA **(A)**, and at BDC, BR10 and RT for OA **(B)**. Data are displayed as mean ± SD values. Asterisks denote significant values (*p<0.05 and ***p<0.001).

### 3.2 Motor units properties

#### 3.2.1 Changes in MU properties in YA

All YA participants completed the assessments successfully. No MUs were detected in participants Y6 and Y5 at BR21 and RT, respectively. In total, 897 MUs were identified from HDsEMG recordings (255 at BDC, 246 at BR21, 396 at RT). From this dataset, 546 units (152 at BDC, 146 at BR21, 248 at RT) and 557 units (163 at BDC, 137 at BR21, 257 at RT) met the inclusion criteria for paired MU analysis and geometric approach, respectively.

Linear mixed models revealed changes in PIC-related indices following 21 days of bed rest and subsequent retraining in YA (**Figure 3**). ΔF (**Figure 3A**) was lower at BR21 than at BDC (26.1%, p < 0.001), did not differ between RT and BDC (p = 1.000), and was higher at RT than at BR21 (21.8%, p < 0.001). BH (**Figure 3C**) did not differ between BR21 and BDC or between RT and BDC but was higher at RT than at BR21 (4.05%rTri, 9.5%, p = 0.015). ACC (**Figure 3E**) was lower at BR21 than at BDC (0.48 pps/%MVC, 25.1%, p = 0.002), did not differ between RT and BDC (p = 1.000), and was higher at RT than at BR21 (32.1%, p < 0.001). Similarly, ATT (**Figure 3G**) was lower at BR21 than at BDC (0.11 pps/%MVC, 27.7%, p = 0.023), did not differ between RT and BDC (p = 0.768), and was higher at RT than at BR21 (49.8%, p < 0.001).

**Figure 3:**
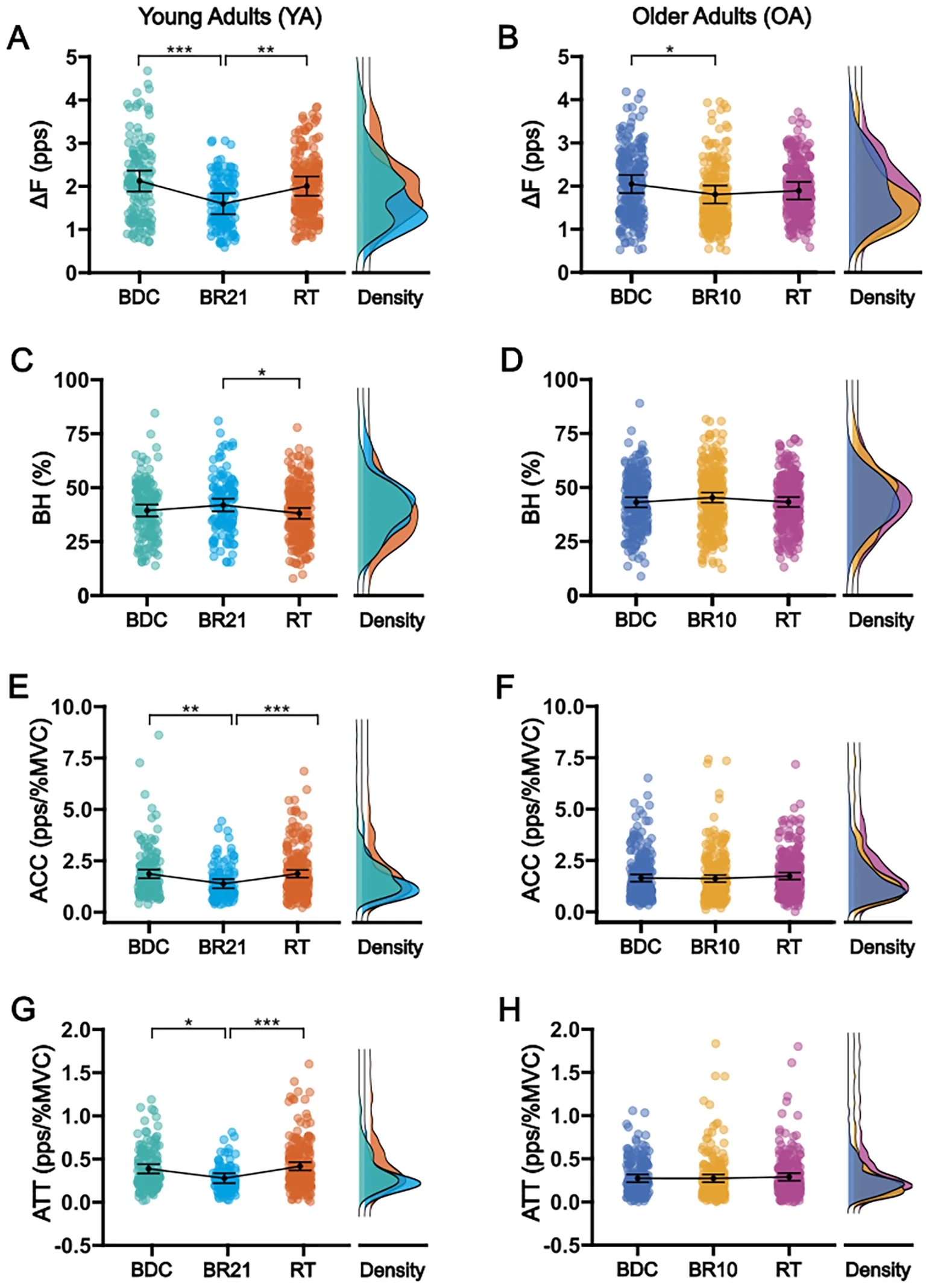
Swarm plots represent delta frequency [ΔF, **(A, B)**], brace height **(C, D)**, acceleration **(E, F)**, and attenuation **(G, H)** at each data collection point in young adults [YA, **(A, C, E, G)**] and older adults [OA, **(B, D, F, H)**] during triangular contractions at 20% of MVC. Solid lines represent estimated marginal means, with upper and lower lines indicating the 95% confidence interval. Each dot refers to the averaged ΔF value per test unit **(A, B)** or individual MU values **(C–H)**. Asterisks denote significant differences (*p<0.05, **p<0.01 and ***p<0.001).

Discharge rate properties also changed after bed rest and retraining in YA (**Figure 4**). Peak discharge rate (**Figure 4A**) was lower at BR21 than at BDC (2.66 pps, 19.4%, p < 0.001), did not differ between RT and BDC (p = 0.179), and was higher at RT than at BR21 (13.4%, p < 0.001). Discharge rate range (**Figure 4C**) was lower at BR21 than at BDC (2.44 pps, 22.9%, p < 0.001), remained lower at RT than at BDC (11.7%, p = 0.031), and was higher at RT than at BR21 (14.8%, p < 0.001). Ascending discharge rate range (**Figure 4E**) was lower at BR21 than at BDC (2.05 pps, 23.5%, p < 0.001), did not differ between RT and BDC (p = 0.064), and was higher at RT than at BR21 (14.7%, p < 0.001). Descending discharge rate range (**Figure 4G**) was lower at BR21 than at BDC (2.08 pps, 22.8%, p < 0.001), remained lower at RT than at BDC (10.1%, p = 0.004), and was higher at RT than at BR21 (13.7%, p < 0.001).

**Figure 4:**
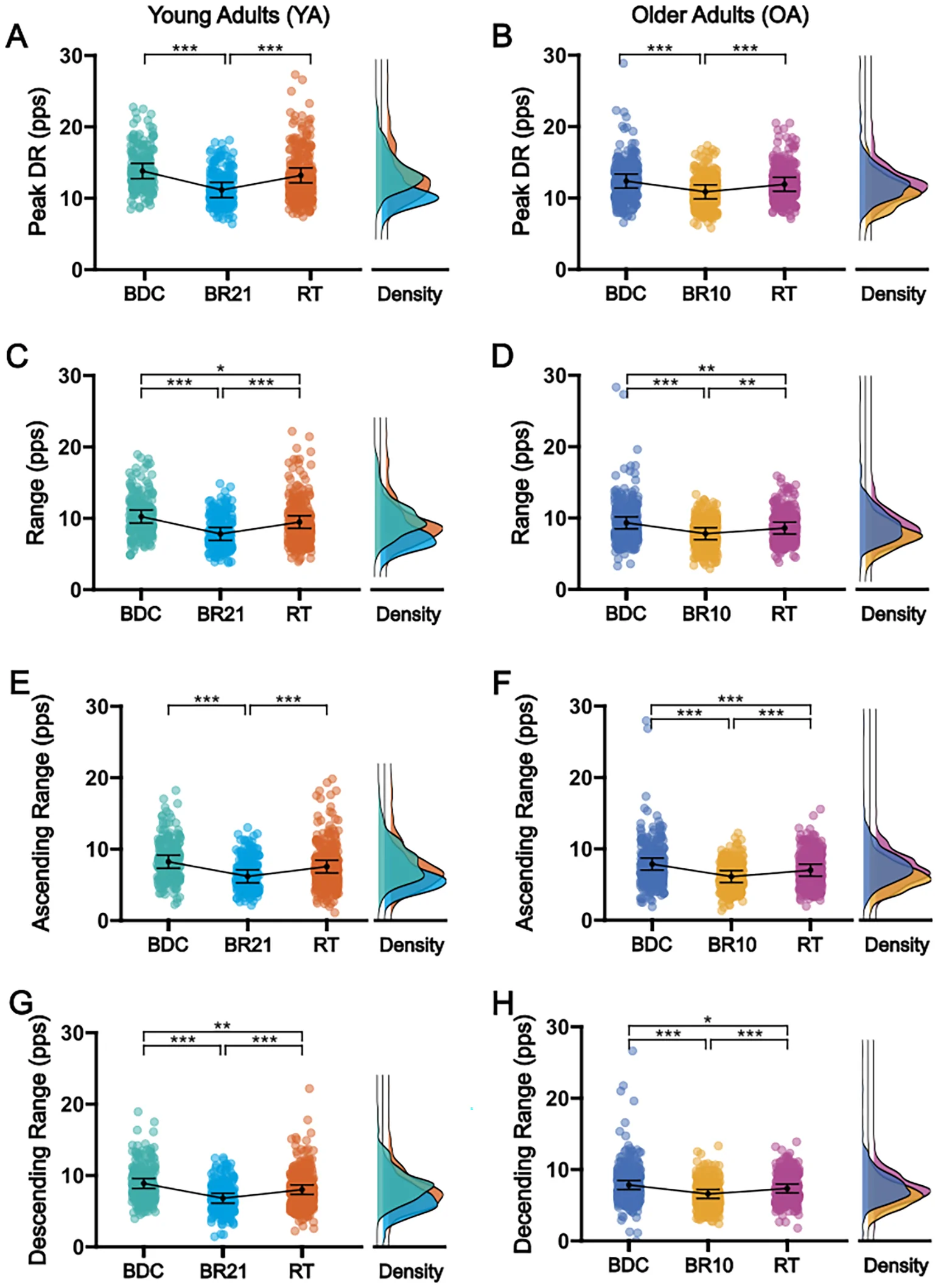
Swarm plots represent peak discharge rate **(A, B)**, discharge rate range **(C, D)**, ascending discharge rate range **(E, F)**, and descending discharge rate range **(G, H)** at each data collection point in young adults [YA, **(A, C, E, G)**] and older adults (OA, **(B, D, F, H)** during triangular contractions at 20% of MVC. Solid lines represent estimated marginal means, with upper and lower lines indicating the 95% confidence interval. Each dot refers to individual MUs. Asterisks denote significant differences (*p<0.05, **p<0.01 and ***p<0.001).

#### 3.2.2 Changes in MU properties in OA

All OA participants completed the assessments successfully. In total, 1179 MUs were identified from HDsEMG recordings (376 at BDC, 394 at BR10, 409 at RT). From this dataset, 785 units (244 at BDC, 261 at BR10, 280 at RT) and 812 units (263 at BDC, 268 at BR10, 281 at RT) met the inclusion criteria for paired MU analysis and geometric approach, respectively.

Linear mixed models revealed changes in indices of PIC contribution to motoneuron discharge following 10 days of bed rest and subsequent recovery in OA (**Figure 3**). ΔF (**Figure 3B**) was lower at BR10 than at BDC (0.25 pps, 15.3%, p=0.029), whereas values after the recovery period did not differ significantly from either baseline (BDC vs RT: p=0.279) or following bed rest (BR10 vs RT: p=0.994). BH, ACC and ATT (**Figures 3D, F, H**) did not change significantly at any time point in OA (all p>0.189).

MU discharge properties in OA decreased following bed rest but showed variable degrees of recovery following retraining (**Figure 4**). Peak discharge rate (**Figure 4B**) was lower at BR10 than at BDC (1.51 pps, 13.0%, p < 0.001), did not differ between RT and BDC (p = 0.173), and was higher at RT than at BR10 (12.1%, p < 0.001). Discharge rate range (**Figure 4D**) was lower at BR10 than at BDC (1.52 pps, 18.1%, p < 0.001), remained lower at RT than at BDC (6.9%, p = 0.003), and was higher at RT than at BR10 (13.6%, p = 0.002). Ascending discharge rate range (**Figure 4F**) was lower at BR10 than at BDC (1.75 pps, 22.9%, p < 0.001), remained lower at RT than at BDC (9.7%, p = 0.001), and was higher at RT than at BR10 (17.1%, p < 0.001). Descending discharge rate range (**Figure 4H**) was lower at BR10 than at BDC (1.24 pps, 18.0%, p < 0.001), remained lower at RT than at BDC (5.8%, p = 0.042), and was higher at RT than at BR10 (14.9%, p < 0.001).

### 3.3 Blood biomarkers

Plasma concentrations of TRP, KYN and TRP/KYN ratio did not change significantly following bed rest or recovery in YA (all p>0.903; **Figures 5A, C, E**). Similarly, no significant changes were observed in OA at any time point (all p>0.279; **Figures 5B, D, F**).

**Figure 5:**
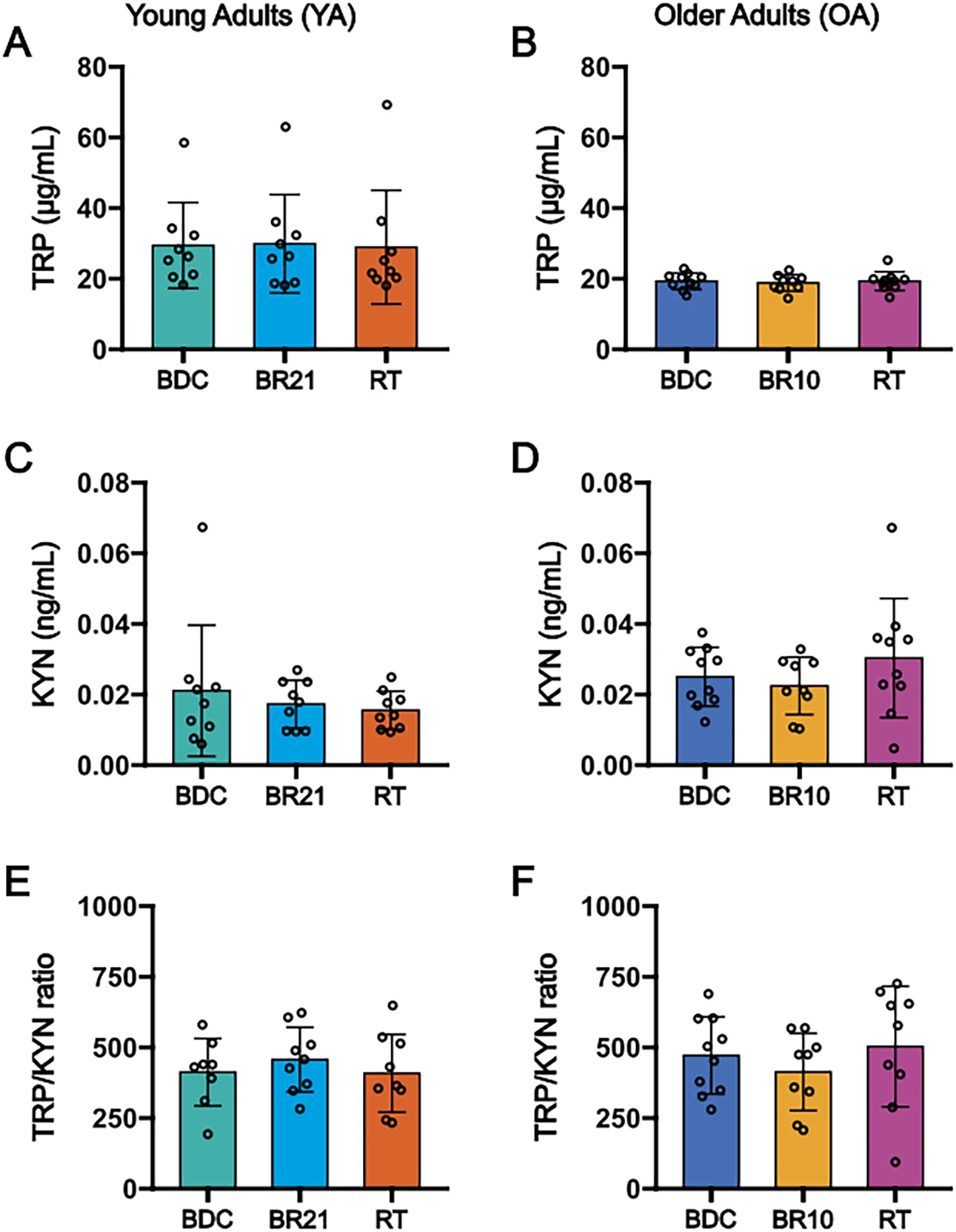
Bar plots illustrate plasma concentrations of Tryptophan [TRP, **(A, B)**], Kynurenine [KYN, **(C, D)**], and Tryptophan/Kynurenine ratio [TRP/KYN, **(E, F)**] at each data collection point in young adults [YA, **(A, C, E)**] and older adults [OA, **(B, D, F)**]. Data are displayed as mean ± SD values.

### 3.4 Supplemental analysis

Because YA and OA underwent different bed rest durations, direct between-group comparisons were considered exploratory and are reported in the **Supplementary Material**. In addition, complementary analyses performed using contractions at 20% of baseline MVC, to account for changes in absolute force across time points, are also provided in the **Supplementary Material**. When the analyses were repeated using contractions performed at the same absolute force across sessions, ΔF still decreased after bed rest and was no longer different from baseline after retraining in both groups, indicating that the main ΔF findings were not dependent on changes in relative contraction intensity.

## 4 Discussion

The present study investigated how prolonged bed rest and subsequent retraining influence motoneuron intrinsic excitability in young and older adults, with a particular focus on PIC/related indices derived from paired MU analysis and a complementary geometric approach. Prolonged bed rest reduced ΔF in young and older males whereas subsequent retraining promoted recovery of this measure. This supports the view that motoneuron excitability is sensitive to inactivity and can be modified by exercise-based recovery. The divergence between ΔF and geometric metrics suggests that these approaches are not interchangeable but instead provide distinctive insights into motoneuron behavior during disuse and recovery. MVC force decreased after bed rest in young adults, showing partial recovery with retraining, but remained unchanged in older adults. Overall, these findings suggest that neural and functional adaptations to disuse and retraining may not proceed in parallel. In particular, MU-level indices appeared sensitive to disuse even when MVC force was unchanged in older adults, and ΔF was no longer different from baseline after retraining despite incomplete MVC recovery in young adults. This apparent dissociation should be interpreted cautiously because bed rest duration differed between groups, but it suggests that neural indices may provide information not captured by maximal force measurements alone.

### 4.1 Changes in persistent inward current estimates after bed rest

The present study provides novel evidence that prolonged bed rest reduces ΔF, an indirect PIC-related estimate, in both young and older males. The observed decreases in ΔF align with our previous findings after 10-days of unilateral lower limb suspension in young adults (Martino et al., 2024), and extend these observations to prolonged whole-body inactivity and to older individuals. MU discharge rate properties mirrored these changes, with decreases in peak discharge rate and discharge rate range after bed rest, consistent with the central role of PICs in amplifying synaptic input and sustaining MU firing (Heckman et al., 2008).A central issue in interpreting these findings is the non-uniform response of ΔF and the geometric discharge metrics. Although ΔF decreased after bed rest in both groups, brace height did not show a consistent reduction. This indicates that ΔF and geometric metrics should not be interpreted as interchangeable estimates of a single physiological mechanism. Simulation work suggests that both ΔF and BH may be influenced by neuromodulatory drive (Beauchamp et al., 2023), but ΔF is also sensitive to excitation-inhibition coupling, whereas BH reflects ascending discharge-rate non-linearity. Recent experimental and simulation work further suggests that ΔF and BH can diverge, with ΔF reflecting discharge prolongation and BH reflecting discharge amplification (Škarabot et al., 2025). In this context, the reduction in ΔF without a consistent reduction in BH may suggest that bed rest affected discharge prolongation or excitation-inhibition coupling differently from ascending discharge-rate amplification. The behavior of ACC and ATT adds further nuance: ACC decreased in young adults, suggesting altered ascending discharge dynamics, whereas ATT did not increase, providing no evidence that enhanced descending-phase attenuation was the primary mechanism underlying the reduction in ΔF. Therefore, the present findings support the use of ΔF, BH, ACC, and ATT as complementary, but non-interchangeable, descriptors of MU discharge behavior.

One possible physiological interpretation is that these metrics differ in their sensitivity to distinct components or time courses of PIC-related discharge behavior. PICs are mediated by both voltage-gated calcium (CaV) and sodium (NaV) channels, which differ in their activation kinetics and contribution to motoneuron firing (Heckman et al., 2005). CaV-mediated currents are generally associated with sustained firing, whereas NaV-mediated currents may contribute more rapidly to the amplification of synaptic input. Within this framework, the divergent behavior of ΔF and geometric metrics may reflect differences in the sensitivity of these estimates to distinct components of PIC-related discharge behavior (Škarabot et al., 2025). However, this interpretation remains speculative, as the present study did not directly measure CaV- or NaV-mediated currents.

Several mechanisms may contribute to the observed changes in PIC-related estimates. A decrease in corticospinal excitation could diminish the excitatory influence on brainstem serotonergic and noradrenergic nuclei that are essential for PIC facilitation (Heckman et al., 2008). In support of this hypothesis, immobilization and prolonged inactivity have been shown to alter motor cortex organization and excitability, potentially leading to hypoexcitability and reduced corticospinal drive (Clark et al., 2006; Langlet et al., 2012; Rosenkranz et al., 2014). Reduced monoaminergic drive may also arise from reduced availability of circulating monoamines. Animal studies have shown that acute immobilization alters central monoamine levels, suggesting a reduction of noradrenaline and serotonin availability (Corrodi et al., 1968; Kumari et al., 2016), though whether similar changes occur in humans following bed rest remains to be established. In addition, altered proprioceptive feedback and the excitability of spinal reflex pathways may represent further contributing factors. Previous studies have reported reductions in spinal reflex excitability following bed rest or limb disuse, potentially involving altered Ia afferent transmission, presynaptic inhibition, or reflex pathway modulation (Yamanaka et al., 1999; Smith et al., 2022). These findings indicate that disuse can affect spinal sensorimotor pathways, although they do not directly identify the inhibitory influences captured by the attenuation metric. In the present study, attenuation did not increase after bed rest, providing no evidence that enhanced descending-phase inhibitory influences were a primary mechanism underlying the reduction in ΔF. Beyond supraspinal, monoaminergic and proprioceptive influences, intrinsic motoneuron properties may also be altered, with evidence suggesting disuse-induced suppression of voltage-gated calcium and sodium channel expression and function (Sun et al., 2015; Banzrai et al., 2016).

Each of these mechanisms is physiologically plausible and supported to varying degrees by existing evidence. It is likely that several of these operate concurrently to reduce PIC estimates during prolonged inactivity (Piasecki, 2025). Further work is needed to determine their relative contributions and to establish whether these effects differ between young and older adults.

### 4.2 Retraining and exploratory age-related context

Formal between-group comparisons are provided in the **Supplementary Material** and should be interpreted cautiously because the bed rest durations differed between groups. In the main analysis, ΔF was reduced after bed rest in both groups and was no longer different from baseline after retraining in both groups. Therefore, the present results support the sensitivity of ΔF to disuse and its modulation after retraining, but do not provide definitive evidence for age-related differences. The exploratory Group × Time analysis reported in the **Supplementary Material** was consistent with this interpretation, as the interaction for ΔF did not reach statistical significance.

In young adults, 21 days of retraining restored ΔF to baseline levels, confirming that PIC-related indices are plastic and responsive to physical activity. This aligns with our previous finding of full recovery in ΔF following training after disuse in young adults (Martino et al., 2024). The geometric approach provided additional context, as the restoration of acceleration and attenuation in young adults was consistent with re-established neuromodulatory and inhibitory influences on MU discharge. Evidence from animal studies suggests that exercise-induced adaptations may encompass multiple mechanisms that could influence PIC estimates: enhanced descending monoaminergic drive to motoneurons (Meeusen and De Meirleir, 1995), increased receptor sensitivity to neuromodulators (Gardiner et al., 2006), and structural changes in motoneuron dendrites that facilitate PIC activation (Krutki et al., 2017). Recent evidence further suggests that chronic exercise may modulate ion channels, promote dendritic plasticity, and increase the expression of excitatory synapses and neurotrophins (Dai et al., 2024). These mechanisms remain plausible but were not directly tested in the present study.

In older adults, ΔF was reduced after bed rest, while values at RT were no longer different from baseline. However, ΔF at RT also did not differ significantly from BR10, and geometric metrics remained unchanged across time. Thus, the post-retraining response in older adults should be interpreted cautiously: although ΔF values at RT were compatible with recovery toward baseline, the absence of a significant BR10-to-RT increase, and the stability of geometric metrics suggest that modulation of PIC-related indices was less clearly resolved under the present protocol. As discussed above, the divergence between ΔF and the geometric metrics suggests that these measures may capture complementary but non-identical aspects of MU discharge behavior.

### 4.3 Maximal voluntary contraction force

Bed rest-induced reductions of MVC in YA are consistent with previous reports of rapid strength loss following disuse, reflecting both peripheral muscular atrophy and central neuromuscular adaptations (Clark, 2009; Monti et al., 2021; Motanova et al., 2026). In OA, MVC did not significantly change, likely due to the shorter bed rest duration. This does not imply older adults are unaffected; instead, the lower disuse exposure may have limited the observable MVC decline. Notably, the absence of MVC decline in OA did not preclude changes in MU discharge properties, including reductions in ΔF and discharge rate characteristics. This dissociation suggests that MU-level indices may detect adaptations to disuse that are not necessarily reflected in maximal force output, particularly after a shorter disuse exposure.

Endurance retraining effectively improved MVC, consistent with prior reports that exercise training can reverse or mitigate disuse-induced muscle weakness (Lee et al., 2010; Vigelsø et al., 2015). However, our young participants did not completely recover to baseline levels. This likely reflects the nature of the retraining: endurance training enhances mitochondrial function and oxidative capacity (Mølmen et al., 2025), but may not provide sufficient mechanical load to restore myofibrillar content, muscle cross-sectional area, or maximal MU recruitment (Wilkinson et al., 2008; Vila-Chã et al., 2010). Thus, while endurance retraining was beneficial, fully reversing bed rest–induced strength loss in young adults may require longer duration, higher mechanical load, or multimodal approaches.

### 4.4 Blood biomarkers

Despite the electrophysiological changes, plasma TRP, KYN, and the TRP/KYN ratio remained stable across bed rest and retraining. These biomarkers were included to provide exploratory systemic context related to tryptophan metabolism, given the role of serotonergic input in the modulation of motoneuron excitability and PIC facilitation (Perrier et al., 2013). However, circulating TRP and KYN should not be interpreted as direct surrogates of central serotonergic drive, spinal monoaminergic activity, or serotonin availability at the motoneuron level (Tikhonova et al., 2022). Plasma concentrations of these metabolites are influenced by several peripheral processes, including diet, liver metabolism, immune activation, and skeletal muscle metabolism. Therefore, the biomarker null result should be interpreted as a negative exploratory finding rather than evidence against central adaptation. Rather, it indicates that the observed PIC-related and MU discharge changes were not accompanied by detectable alterations in these systemic biomarkers.

## 5 Limitations

Several limitations of this study should be acknowledged. First, the duration of bed rest differed between young and older adults (21 vs. 10 days) for safety and ethical reasons, which limits direct age comparisons and requires caution when interpreting differences between groups. Second, MUs were not tracked across experimental sessions; therefore, the present results reflect population-level adaptations rather than longitudinal changes within the same identified units. This reflects the methodological challenge of reliably identifying the same MUs across different recording sessions. Third, contractions were performed at low force (20% MVC) to facilitate accurate MU decomposition, and PIC-related behavior at higher contraction intensities may differ. The apparent dissociation between MVC and MU-level indices should also be interpreted cautiously, as MVC was assessed during maximal contractions, whereas MU discharge and PIC-related estimates were obtained at 20% MVC. However, 20% MVC approximates the level of muscle activation typically required for daily life activities such as walking, supporting the functional relevance of the present findings. Fourth, only male participants were included, in accordance with the ethics committee approval for this bed-rest protocol, limiting generalizability to females. Fifth, the study did not include direct measures of spinal inhibitory circuits, corticospinal drive or central monoaminergic activity, which would have provided deeper mechanistic insight into the observed reductions in PIC indices. Sixth, potential bed rest-induced musculoskeletal changes were not assessed within the present analysis; therefore, their influence on proprioceptive feedback and MU discharge behavior cannot be excluded. Finally, because contractions were performed at 20% MVC, the present findings primarily reflect low-threshold MU behavior. PIC-related adaptations may differ at higher contraction intensities, where higher-threshold MUs are recruited. Future studies should determine whether similar adaptations occur at higher force levels and in higher-threshold motor units, which may be more directly relevant to maximal force production and high-demand functional tasks.

## 6 Conclusions

Prolonged bed rest reduced ΔF in both young and older males, consistent with reduced motoneuron excitability after disuse. Following retraining, ΔF was no longer different from baseline, supporting the plasticity of this PIC-related estimate after inactivity. In young adults, disuse and recovery were also accompanied by changes in geometric metrics, whereas these indices remained unchanged in older adults under the present protocol. Together, these results indicate that PIC-related adaptations to disuse and retraining are not uniform across metrics, suggesting that distinct neural mechanisms may contribute to changes in ΔF and geometric discharge features in young and older individuals. The absence of changes in circulating TRP and KYN despite clear electrophysiological alterations emphasizes the complex relationship between peripheral markers and central neural adaptations. Future research should combine motor-unit analyses with direct measures of spinal and monoaminergic function (Bjorefeldt et al., 2018), and compare different training modalities or pharmacological interventions for restoring motoneuron excitability across age groups.

## Data Availability

The raw data supporting the conclusions of this article will be made available by the authors, without undue reservation.
