# Supplementary material for: Bed rest and endurance retraining alter persistent inward current estimates in young and older males
Source: Front Physiol. 2026 Sep 22;17:1905824. doi: 10.3389/fphys.2026.1905824 (PMC13638420; doi:10.3389/fphys.2026.1905824)
Supplement: Supplementary file 1 [file Supplementaryfile1.docx]

**Supplementary Material**

This Supplementary Material provides additional analyses to support and extend the main findings. Specifically, it includes: (i) direct statistical comparisons between young and older adults across time points, and (ii) analyses performed using contractions normalized to baseline MVC to account for changes in absolute force across time points.

**Age-related differences**

Age-related differences were assessed using an additional linear mixed model with group (YA vs OA), time (BDC, BR, RT) and their interaction (Group × Time) as fixed effect. Pairwise contrasts between groups were estimated at each time point (BDC, BR and RT) using estimated marginal means with Bonferroni correction for multiple comparisons. However, given that the duration of bed rest differed between YA (21 days) and OA (10 days), group comparisons at the bed rest time point should be interpreted with caution, as they reflect differences following interventions of unequal length.

Significant main effects of Time were observed for ΔF (p < 0.001), BH (p = 0.007), ACC (p < 0.001), ATT (p < 0.001), peak discharge rate (p < 0.001), discharge rate range (p < 0.001), ascending discharge rate range (p < 0.001), descending discharge rate range (p < 0.001), and MVC (p = 0.030). Significant main effects of Group were found for BH (p = 0.011), ATT (p = 0.005), MVC (p = 0.001), TRP/KYN ratio (p = 0.015), and TRP (p < 0.001). Significant Group × Time interactions emerged for ACC (p = 0.017), ATT (p = 0.008), peak discharge rate (p = 0.012), and discharge rate range (p = 0.030), indicating that the pattern across BDC, BR, and RT differed between young and older participants for these variables. No significant interaction was observed for ΔF (p = 0.057), BH (p = 0.583), MVC (p = 0.088), TRP/KYN ratio (p = 0.315), KYN (p = 0.349), TRP (p = 0.975), ascending discharge rate range (p = 0.447), or descending discharge rate range (p = 0.071). KYN showed no significant main effect of session (p = 0.914), group (p = 0.365), or interaction (p = 0.349), whereas MVC showed significant main effects of both session and group in the absence of a significant interaction. Group comparisons are reported in Table 1.

*Force assessment.* At BDC, YA displayed significantly higher MVC compared to OA (191.9 N, p=0.001), while no significant group differences were observed at BR (23.97 N, p=0.650) or RT (97.60 N, p=0.077).

*Intrinsic motoneuron properties.* No significant group differences were observed for ΔF at any time point (all p>0.450). BH was significantly lower in YA compared to OA at BDC (3.68%rTri, p=0.047) and RT (5.14%rTri, p=0.005), but not at BR (p=0.079). ACC did not differ between groups at any time point (all p>0.099). ATT was significantly higher in YA compared to OA at BDC (0.11 pps, p=0.002) and RT (0.13 pps, p=0.003), while no group difference was observed at BR (p=0.890).

*Discharge rate characteristics.* No significant group differences were observed for peak discharge rate, discharge rate range or ascending discharge rate range at any time point (all p>0.076). Descending discharge rate range was significantly higher in YA compared to OA at BDC (1.05 pps, p=0.029), but not at BR (p=0.620) or RT (p=0.140).

*Blood biomarkers.* TRP was significantly higher in YA compared to OA at all time points (BDC: 10.14 µg/mL, p=0.037; BR: 11.21 µg/mL, p=0.023; RT: 9.62 µg/mL, p=0.047). No significant group differences were observed for KYN at any time point (all p>0.130). TRP/KYN ratio did not differ between groups at BDC or BR (all p>0.240), but was significantly lower in YA compared to OA at RT (-0.02, p=0.011).

**Changes in MU properties in YA at same absolute force across sessions**

Three additional triangular contractions at 20% of baseline MVC were collected post-bed rest and post-retraining to account for possible changes in absolute force.

In total, 787 MUs were identified from HDsEMG recordings at the absolute force level (255 at BDC, 230 at BR21, 302 at RT). From this dataset, 475 units (152 at BDC, 134 at BR21, 189 at RT) and 489 units (163 at BDC, 147 at BR21, 179 at RT) met the inclusion criteria for paired motor unit analysis and geometric approach, respectively.

Linear mixed models revealed changes in intrinsic motoneuron properties following 21 days of bed rest and subsequent recovery in YA at the absolute force level (Supplemental Fig. 1). ΔF (Supplemental Fig. 1A) decreased at BR21 compared to BDC (28.1%, p<0.001), while the recovery period restored initial values, with ΔF increasing from BR21 (37.1%, p<0.001; BDC vs RT: p=1.000). BH (Supplemental Fig. 1C) did not change significantly following bed rest or recovery in YA, although a significant increase was observed from BR21 to RT (4.61%rTri, 10.9%, p=0.004). ACC (Supplemental Fig. 1E) decreased following bed rest compared to BDC (0.50 pps, 25.7%, p<0.001), and was restored during recovery, with values at RT returning to baseline levels (BR21 vs RT: 29.5%, p=0.004; BDC vs RT: p=1.000). Similarly, ATT (Supplemental Fig. 1G) decreased following bed rest (0.14 pps, 34.2%, p<0.001) and was restored at RT (BR21 vs RT: 48.0%, p<0.001; BDC vs RT: p=1.000).

Discharge rate properties also changed post-bed rest and recovery in YA at the absolute force level (Supplemental Fig. 2). Peak discharge rate (Supplemental Fig. 2A) decreased following bed rest compared to BDC (2.54 pps, 16.9%, p<0.001) and was fully restored during recovery, returning to baseline levels at RT (BR21 vs RT: 20.2%, p<0.001; BDC vs RT: p=1.000). Similarly, discharge rate range (Supplemental Fig. 2C) decreased at BR21 compared to BDC (2.35 pps, 20.6%, p<0.001), and was fully restored at RT (BR21 vs RT: 24.6%, p<0.001; BDC vs RT: p=1.000). Ascending discharge rate range (Supplemental Fig. 2E) decreased following bed rest (1.92 pps, 20.7%, p<0.001) and was fully restored at RT (BR21 vs RT: 23.3%, p<0.001; BDC vs RT: p=1.000). Descending discharge rate range (Supplemental Fig. 2G) also decreased at BR21 (2.00 pps, 20.6%, p<0.001), with values fully restored at RT (BR21 vs RT: 23.1%, p<0.001; BDC vs RT: p=1.000).

**Changes in MU properties in OA at same absolute force across sessions**

Three additional triangular contractions at 20% of baseline MVC were collected post-bed rest and post-retraining to account for possible changes in absolute force.

In total, 1187 MUs were identified from HDsEMG recordings at the absolute force level (376 at BDC, 399 at BR10, 412 at RT). From this dataset, 778 units (244 at BDC, 253 at BR10, 281 at RT) and 814 units (263 at BDC, 262 at BR10, 289 at RT) met the inclusion criteria for paired motor unit analysis and geometric approach, respectively.

Linear mixed models revealed changes in intrinsic motoneuron properties following 10 days of bed rest and subsequent recovery in OA at the absolute force level (Supplemental Fig. 1). ΔF (Supplemental Fig. 1B) was lower at BR10 than at BDC (0.27 pps, 13.3%, p = 0.016), whereas values at RT did not differ from either BDC (p = 1.000) or BR10 (p = 0.070). BH, ACC and ATT (Supplemental Figs. 1D, 1F, 1H) did not change significantly at any time point in OA (all p>0.999).

Motor unit discharge properties in OA decreased following bed rest but showed variable degrees of recovery following retraining at the absolute force level (Supplemental Fig. 2). Peak discharge rate (Supplemental Fig. 2B) decreased following bed rest compared to BDC (1.25 pps, 9.8%, p<0.001) and was fully restored during recovery, returning to baseline levels at RT (BR10 vs RT: 10.2%, p<0.001; BDC vs RT: p=1.000). Similarly, discharge rate range (Supplemental Fig. 2D) decreased at BR10 compared to BDC (1.24 pps, 13.4%, p<0.001), and was fully restored at RT (BR10 vs RT: 12.5%, p<0.001; BDC vs RT: p=0.548). Ascending discharge rate range (Supplemental Fig. 2F) decreased following bed rest (1.36 pps, 17.2%, p<0.001) and was partially restored at RT (BR10 vs RT: 13.4%, p=0.013; BDC vs RT: 6.2%, p=0.028). Descending discharge rate range (Supplemental Fig. 2H) also decreased at BR10 (1.10 pps, 14.1%, p<0.001), with values fully restored at RT (BR10 vs RT: 14.5%, p<0.001; BDC vs RT: p=1.000).

| **Variable** | **Contrast** | **Estimate** | **SE** | **df** | **t** | **p (adj)** | **95% CI** |
| --- | --- | --- | --- | --- | --- | --- | --- |
| MVC (N) | YA vs OA BDC | 191.90 | 52.76 | 22 | 3.64 | **0.001** | [82.64 301.15] |
|  | YA vs OA BR | 23.97 | 52.76 | 22 | 0.45 | 0.65 | [-85.28 133.22] |
|  | YA vs OA RT | 97.60 | 52.76 | 22 | 1.85 | 0.077 | [-11.66 206.85] |
| ΔF (pps) | YA vs OA BDC | 0.08 | 0.16 | 31 | 0.48 | 0.63 | [-0.25 0.40] |
|  | YA vs OA BR | -0.21 | 0.16 | 32 | -1.3 | 0.19 | [-0.53 0.11] |
|  | YA vs OA RT | 0.11 | 0.15 | 24 | 0.77 | 0.45 | [-0.19 0.42] |
| BH (%) | YA vs OA BDC | -3.68 | 1.80 | 38 | -2.05 | **0.047** | [-7.32 -0.05] |
|  | YA vs OA BR | -3.34 | 1.86 | 43 | -1.80 | 0.079 | [-7.09 0.41] |
|  | YA vs OA RT | -5.14 | 1.67 | 28 | -3.07 | **0.005** | [-8.57 -1.71] |
| ACC (pps) | YA vs OA BDC | 0.21 | 0.14 | 64 | 1.57 | 0.12 | [-0.06 0.49] |
|  | YA vs OA BR | -0.24 | 0.14 | 73 | -1.67 | 0.099 | [-0.52 0.05] |
|  | YA vs OA RT | 0.13 | 0.12 | 44 | 1.01 | 0.32 | [-0.13 0.38] |
| ATT (pps) | YA vs OA BDC | 0.11 | 0.04 | 60 | 3.23 | **0.002** | [0.04 0.18] |
|  | YA vs OA BR | 0.005 | 0.04 | 66 | 0.14 | 0.89 | [-0.07 0.08] |
|  | YA vs OA RT | 0.13 | 0.03 | 41 | 3.94 | **0.003** | [0.06 0.19] |
| Peak DR (pps) | YA vs OA BDC | 1.44 | 0.70 | 22 | 2.05 | 0.051 | [-0.01 2.90] |
|  | YA vs OA BR | 0.30 | 0.70 | 22 | 0.43 | 0.67 | [-1.16 1.76] |
|  | YA vs OA RT | 1.29 | 0.69 | 21 | 1.87 | 0.076 | [-0.15 2.72] |
| Range (pps) | YA vs OA BDC | 0.91 | 0.60 | 24 | 1.52 | 0.14 | [-0.32 2.14] |
|  | YA vs OA BR | 0.003 | 0.60 | 24 | 0.006 | 0.99 | [-1.23 1.24] |
|  | YA vs OA RT | 0.89 | 0.59 | 22 | 1.52 | 0.14 | [-0.33 2.10] |
| Asc Range (pps) | YA vs OA BDC | 0.37 | 0.60 | 24 | 0.61 | 0.55 | [-0.88 1.61] |
|  | YA vs OA BR | 0.07 | 0.60 | 24 | 0.11 | 0.91 | [-1.18 1.31] |
|  | YA vs OA RT | 0.54 | 0.59 | 22 | 0.92 | 0.37 | [-0.68 1.76] |
| Des Range (pps) | YA vs OA BDC | 1.05 | 0.46 | 27 | 2.30 | **0.029** | [0.11 1.99] |
|  | YA vs OA BR | 0.23 | 0.46 | 27 | 0.51 | 0.62 | [-0.71 1.17] |
|  | YA vs OA RT | 0.67 | 0.44 | 23 | 1.52 | 0.14 | [-0.24 1.58] |
| TRP (µg/mL) | YA vs OA BDC | 10.14 | 4.54 | 19 | 2.23 | **0.037** | [0.65 19.63] |
|  | YA vs OA BR | 11.21 | 4.55 | 20 | 2.46 | **0.023** | [1.70 20.73] |
|  | YA vs OA RT | 9.62 | 4.53 | 19 | 2.12 | **0.047** | [0.13 19.11] |
| KYN (ng/mL) | YA vs OA BDC | -67.95 | 69.39 | 32 | -0.98 | 0.33 | [-209.31 73.41] |
|  | YA vs OA BR | 30.59 | 69.27 | 32 | 0.44 | 0.66 | [-110.59 171.77] |
|  | YA vs OA RT | -107.96 | 69.27 | 32 | -1.56 | 0.13 | [-249.14 33.22] |
| TRP/KYN r | YA vs OA BDC | -0.004 | 0.006 | 36 | -0.71 | 0.48 | [-0.02 0.007] |
|  | YA vs OA BR | -0.007 | 0.006 | 37 | -1.20 | 0.24 | [-0.02 0.005] |
|  | YA vs OA RT | -0.02 | 0.006 | 36 | -2.67 | **0.011** | [-0.03 -0.004] |

**Supplemental Table 1.** Group differences between young (YA) and older adults (OA) at each time point estimated from linear mixed models. Estimates represent the mean difference (YA − OA) with positive values indicating higher values in YA. SE, standard error; df, degrees of freedom; t, t-ratio; p (adj), p-value adjusted for multiple comparisons using Bonferroni correction; 95% CI, 95% confidence interval. BDC, baseline data collection; BR, bed rest; RT, retraining; MVC, maximum voluntary contraction; BH, brace height; ACC, acceleration; ATT, attenuation; FR, discharge rate; TRP, tryptophan; KYN, kynurenine. Bold values indicate statistical significance (p < 0.05).

**Supplemental Figure 1.** Swarm plots represent delta frequency (ΔF, **A-B**), brace height (BH, **C-D**), acceleration (ACC, **E-F**), and attenuation (ATT, **G-H**) at each data collection point in young adults (YA, **A-C-E-G**) and older adults (OA, **B-D-F-H**) during triangular contractions performed at the same absolute force level (20% of baseline MVC). Solid lines represent estimated marginal means, with upper and lower lines indicating the 95% confidence interval. Each dot refers to the averaged ΔF value per test unit (**A-B**) or individual MUs values (**C-H**). Asterisks denote significant differences (* p<0.05, ** p<0.01 and *** p<0.001).

**Supplemental Figure 2.** Swarm plots represent peak discharge rate (**A-B**), discharge rate range (**C-D**), ascending discharge rate range (**E-F**), and descending discharge rate range (**G-H**) at each data collection point in young adults (YA, **A-C-E-G**) and older adults (OA, **B-D-F-H**) during triangular contractions performed at the same absolute force level (20% of baseline maximal voluntary contraction). Solid lines represent estimated marginal means, with upper and lower lines indicating the 95% confidence interval. Each dot refers to individual MUs. Asterisks denote significant differences (* p<0.05, ** p<0.01 and *** p<0.001).
